# Exogenous 24-epibrassinolide enhances chilling tolerance in *Phalaenopsis-type Dendrobium* through coordinated physiological and transcriptomic responses

**DOI:** 10.3389/fpls.2026.1912617

**Published:** 2026-09-18

**Authors:** Xiaoyun Yu, Xinjie Wei, Tongyan Yao, Xiaoyan Luo, Shuangshuang Yi, Yi Liao, Chonghui Li, Shunjiao Lu

**Affiliations:** 1 Tropical Crops Genetic Resources Institute, Chinese Academy of Tropical Agricultural Sciences, Haikou, China; 2 Key Laboratory of Crop Gene Resources and Germplasm Enhancement in Southern China, Ministry of Agriculture, Haikou, China; 3 Key Laboratory of Tropical Crops Germplasm Resources Genetic Improvement and Innovation of Hainan Province, the Engineering Technology Research Center of Tropical Ornamental Plant Germplasm Innovation and Utilization, Haikou, China; 4 National Key Laboratory for Tropical Crop Breeding, Sanya Research Institute, Chinese Academy of Tropical Agricultural Sciences, Sanya, China

**Keywords:** 24-epibrassinolide (EBR), chilling stress, *Phalaenopsis-type Dendrobium*, transcriptomic analysis, WGCNA

## Abstract

**Introduction:**

*Phalaenopsis-type Dendrobium* is a commercially important tropical orchid that is highly sensitive to chilling, which can cause premature leaf senescence and abscission.

**Methods:**

We evaluated the protective effect of exogenous 24-epibrassinolide (EBR) and integrated time-resolved RNA-seq with physiological traits previously characterized in the same experimental framework. A concentration of 2.0 mg/L was selected for transcriptomic analysis based on the refined dose-response experiment. WGCNA was performed using 18 EBR-treated libraries. Global GO and KEGG outputs were retained as descriptive overviews, whereas quantitative functional inference was based on module-specific analyses with complete multiple-testing-adjusted statistics.

**Results:**

EBR delayed leaf yellowing and abscission. Differential-expression analysis revealed time-dependent transcriptional responses during chilling and recovery. WGCNA identified trait-associated modules with contrasting temporal relationships to physiological traits: the pink module was positively associated with MDA and proline and negatively associated with chlorophyll, whereas the antiquewhite4 (AW4) module showed the opposite pattern. Pink-module enrichment emphasized central carbon and energy metabolism, lipid turnover, peroxisomal processes, autophagy, and cellular transport/homeostasis. AW4 GO enrichment was dominated by transmembrane and metabolite transport; no AW4 KEGG pathway remained significant after multiple-testing correction.

**Discussion:**

These results support an association between EBR treatment and coordinated metabolic, membrane-homeostasis, and stress-response adjustments during chilling and recovery in *Phalaenopsis-type Dendrobium*.

## 1 Introduction

*Dendrobium* is a tropical epiphytic orchid valued for both its ornamental appeal and medicinal properties. Widely cultivated across Southeast Asia for use as cut flowers and potted plants, it also serves as an important source of bioactive compounds such as alkaloids, sesquiterpenes, and polysaccharides with demonstrated immunomodulatory, neuroprotective, and anticancer activities. However, its narrow temperature adaptability—particularly its high sensitivity to chilling temperatures (0–15 °C)—substantially limits its cultivation range, especially in regions prone to fluctuating or suboptimal temperatures (Zhan et al., 2025). Exposure to low temperatures causes membrane destabilization, oxidative damage, and metabolic disruption, resulting in leaf abscission, growth inhibition, and heightened susceptibility to pathogens, thereby threatening both its ornamental and medicinal value.

Cold stress in plants is generally categorized into chilling (0–15 °C) and freezing (<0 °C) stress. In chilling-sensitive species like *D*. phalaenopsis, damage occurs even in the absence of ice formation, emphasizing the detrimental impact of non-freezing low-temperature stress (Banerjee et al., 2017). Chilling stress compromises membrane integrity, impairs photosynthesis, disrupts water and ion homeostasis, and leads to excessive accumulation of reactive oxygen species (ROS), including superoxide anions (O_2_^-^) and hydrogen peroxide (H_2_O_2_) (Ji et al., 2024). These ROS can trigger lipid peroxidation, protein oxidation, and DNA damage if not efficiently detoxified. In response, plants activate antioxidant defense systems comprising enzymes such as superoxide dismutase (SOD), peroxidase (POD), and catalase (CAT), along with the accumulation of compatible solutes such as proline, soluble sugars, and soluble proteins to help maintain osmotic balance and cellular homeostasis (Rajput et al., 2021).

Exogenous application of plant growth regulators has emerged as an effective strategy to mitigate the negative effects of cold stress. Among them, brassinosteroids (BRs)—a class of polyhydroxysteroids recognized as essential phytohormones—play pivotal roles in modulating plant growth, development, and abiotic stress responses. BRs regulate various physiological processes, including cell elongation, vascular differentiation, and photomorphogenesis (Nolan et al., 2020). Notably, BRs have demonstrated significant roles in enhancing cold tolerance by reinforcing the antioxidant defense system, stabilizing cellular membranes, and modulating the expression of stress-responsive genes through the BR signaling pathway (Yamagami et al., 2017; Li et al., 2025b).

24-epibrassinolide (EBR), a highly active and naturally occurring BR analog, has been widely used in exogenous applications due to its high efficacy at nanomolar concentrations (Khan et al., 2022). Extensive studies in various plant species—including *Arabidopsis thaliana*, *Brassica napus*, *Solanum lycopersicum*, and *Vaccinium corymbosum*—have demonstrated that EBR can enhance cold tolerance by activating enzymatic antioxidants (SOD, POD, CAT) (Li et al., 2016), boosting the ascorbate-glutathione (AsA-GSH) cycle, alleviating ROS-induced oxidative damage, enhancing the accumulation of osmoprotectants (e.g., proline, soluble sugars, soluble proteins) (Fan et al., 2025), and upregulating cold-responsive genes such as *CBF*/*DREB* transcription factors (Dou et al., 2021; dos Santos et al., 2023). These multifaceted protective effects underscore that EBR does not operate through a single mechanism, but rather through a complex and highly coordinated network of physiological and molecular responses (Dong et al., 2025).

Given the consistently positive effects of EBR on cold tolerance in diverse crops—including blueberry, tea, peanut, grape, litchi, and peach (Fan et al., 2025)—and the conservation of its underlying mechanisms across plant taxa, it is highly plausible that EBR may also exert protective roles in tropical orchids like *D*. phalaenopsis. Because *D*. phalaenopsis undergoes similar chilling-induced perturbations, including membrane damage and ROS accumulation, EBR application is highly anticipated to trigger analogous mitigating effects. This well-documented, broad-spectrum efficacy of EBR across diverse species establishes a strong empirical basis for extending its use to *Dendrobium*, shifting the hypothesis from a theoretical extrapolation to an evidence-guided inquiry.

Moreover, foliar spraying represents a rapid, efficient, and targeted delivery method for EBR, particularly suitable for temperature-sensitive species like *D.* phalaenopsis, which can respond quickly to environmental changes. Despite accumulating evidence supporting EBR’s effectiveness in enhancing cold tolerance, no systematic studies have been conducted to evaluate its roles in *Dendrobium*.

Therefore, in this study, we evaluated the effects of exogenous foliar EBR on chilling-associated leaf senescence in *Dendrobium* and integrated previously reported physiological measurements from the same experimental framework with time-resolved RNA-seq and WGCNA. Specifically, we characterized EBR-responsive differential expression during chilling and recovery, identified trait-associated co-expression modules, and interpreted their functions using module-specific GO/KEGG enrichment analyses with multiple-testing-adjusted statistics. We also assessed whether the selected EBR treatment produced comparable visible responses across several *Dendrobium* cultivar-by-developmental-stage combinations. This design was intended to define EBR-associated physiological and transcriptional patterns rather than to establish a single causal mechanism.

## 2 Materials and methods

### 2.1 Plant materials, growth conditions, and cold stress treatments

Five-month-old seedlings of the chilling-sensitive *Phalaenopsis-type Dendrobium* cultivar *Den.* ‘Sweel Maple’ were used. Healthy plants with comparable vigor, approximately 10–15 cm in height, were selected to reduce developmental variation among treatments. Plants were maintained under controlled conditions before treatment.

EBR concentration optimization was conducted in two stages. In the preliminary active-agent screen, melatonin (0, 50, 100, 150, and 200 μmol/L), zeaxanthin (0, 20, 40, 60, and 80 mg/L), EBR (0, 0.01, 0.1, 1.0, and 1.5 mg/L), jasmonic acid (0, 50, 100, 200, and 400 μmol/L), proline (0, 5, 10, 15, and 30 mmol/L), and 5-aminolevulinic acid (0, 5, 10, 20, and 40 mg/L) were evaluated; **Figure 1** presents the EBR concentration subset from this screen. Based on the preliminary response, zeaxanthin and EBR were selected for further concentration optimization. The refined EBR screen evaluated 0, 0.1, 0.5, 1.0, 1.5, and 2.0 mg/L, and 2.0 mg/L was identified as the most effective concentration in our previous physiological study (Wei et al., 2025). The 2.0 mg/L treatment was therefore used for the transcriptomic experiment. For the refined treatment, 20.0 mL solution was applied per pot once daily for three consecutive days, followed by a 2-d interval before chilling. Plants were exposed to 5 °C under a 14 h light/10 h dark photoperiod, a light intensity of approximately 20,000 lx, and 85% relative humidity for 8 d, followed by recovery at 25 °C for 7 d. For RNA-seq, samples were collected at 0, 2, 4, and 6 d during chilling and at 9 and 13 d, corresponding to the first and fifth days after transfer to recovery conditions, respectively. Fully expanded apical leaves were harvested, immediately frozen in liquid nitrogen, and stored at -80 °C.

**Figure 1:**
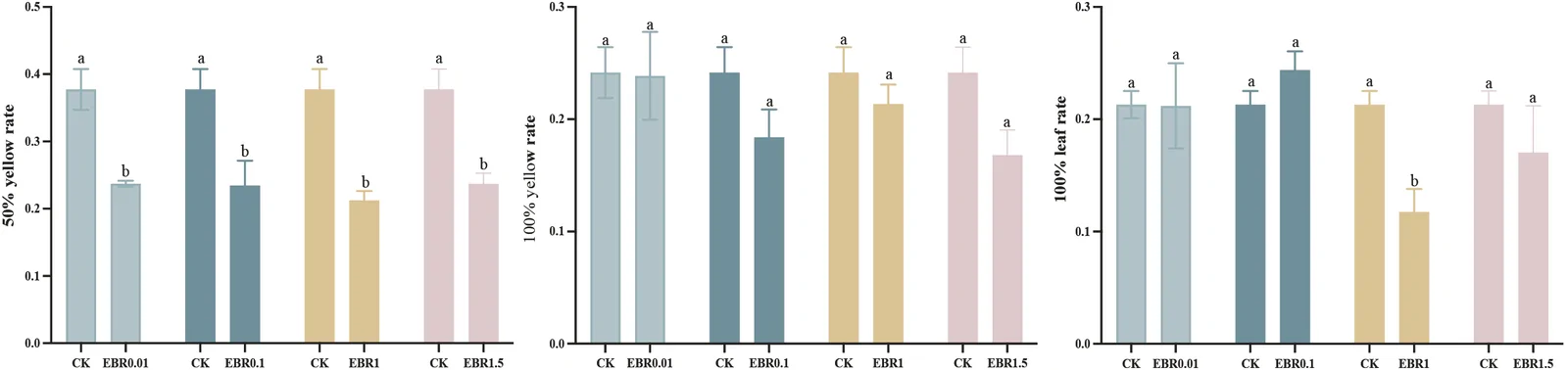
Concentration-dependent effects of EBR in the preliminary screen. Bars show the 50% yellow-leaf rate, 100% yellow-leaf rate, and complete leaf-abscission rate for CK and 0.01, 0.1, 1.0, and 1.5 mg/L EBR treatments. Different lowercase letters indicate significant differences at P < 0.05 according to the statistical analysis described in Materials and Methods.

Each treatment included 12 plants and was evaluated with three biological replicates. Physiological sampling was performed independently from the RNA-seq library preparation. For biochemical measurements, fully expanded leaves were sampled at the corresponding physiological time points; relative electrolyte conductivity was evaluated using five plants per treatment, with three replicate measurements. Data are reported as mean ± standard deviation (SD).

### 2.2 Physiological measurements and leaf-senescence scoring

Leaf yellowing was scored as 50% yellow when approximately 25-75% of the leaf area was yellow and as 100% yellow when more than 75% was yellow; leaves with less than 25% yellow area were not counted as yellow. Defoliated leaves were recorded cumulatively. The physiological measurements used as external WGCNA traits were generated in the same experimental framework and were reported previously (Wei et al., 2025). Briefly, MDA was determined by the thiobarbituric acid method, proline by the acid-ninhydrin method, and total chlorophyll by acetone extraction. In the present study, these previously published measurements were reused only for integrative module-trait association analysis and are not presented as newly generated physiological results.

### 2.3 RNA extraction, library construction, and transcriptome sequencing

Total RNA was extracted from frozen leaf samples using the RNAprep Pure Plant Kit (Tiangen, Beijing, China) according to the manufacturer’s instructions. RNA concentration and purity were assessed using a NanoDrop 2000 spectrophotometer (Thermo Scientific, USA), and RNA integrity was evaluated using agarose gel electrophoresis and an Agilent 2100 Bioanalyzer (Agilent Technologies, USA).

High-quality RNA was used for cDNA library construction with the Illumina TruSeq RNA Sample Preparation Kit according to the manufacturer’s protocol, followed by paired-end sequencing on an Illumina NovaSeq 6000 platform. The present manuscript analyzes only the CK and EBR treatments at six sampling times (0, 2, 4, 6, 9, and 13 d), with three independent biological replicates for each treatment-time combination, resulting in 36 RNA-seq libraries.

### 2.4 Transcriptome data analysis and validation

Raw sequencing reads were quality assessed using FastQC v0.11.5. Adapter sequences and low-quality reads were removed using cutadapt v1.11 with a minimum retained read length of 20 nt, a maximum N fraction of 0.05, and a quality cutoff of 20. Clean reads were aligned to the *Dendrobium* catenatum reference genome assembly ASM160598v2 (NCBI accession GCF_001605985.2) using HISAT2 v2.0.1-beta. For each library, raw reads, clean reads, clean-read ratio, Q20/Q30, GC content, and reference-genome alignment statistics were recorded. Complete quality-control and mapping statistics for all 36 CK/EBR libraries are provided in **Supplementary Table S1**. Because most libraries showed relatively low alignment rates and the three CK13d libraries showed extreme mapping failure, these issues are explicitly reported and considered when interpreting the results.

Gene-level read counts were quantified using featureCounts v1.6.0, and FPKM values were calculated for expression visualization and descriptive comparison. Differential expression between EBR and CK at matched sampling times was analyzed using the edgeR package in R on gene-level count data. Genes with |log2FC| > 1 and a false discovery rate (FDR) < 0.05 were considered differentially expressed genes (DEGs). Comparisons involving CK at 13 d were not used for strong biological inference because of the anomalous mapping performance of the three CK13d libraries.

### 2.5 GO and KEGG enrichment analyses

GO and KEGG enrichment analyses were performed separately for genes assigned to the trait-associated WGCNA modules using the annotation background from the original analysis pipeline. The archived enrichment outputs were generated with a clusterProfiler-based over-representation workflow and include raw P values and multiple-testing-adjusted q values. In the present revision, q < 0.05 was used as the criterion for statistically significant enrichment. Complete quantitative module-specific summaries are provided for pink-module GO (**Supplementary Table S2**), AW4-module GO (**Supplementary Table S3**), pink-module KEGG (**Supplementary Table S4**), and nominal AW4-module KEGG results (**Supplementary Table S5**). AW4 pathways with raw P < 0.05 but q ≥ 0.05 are reported only as nominal signals and are not interpreted as statistically significant.

### 2.6 qRT-PCR validation

To assess concordance between RNA-seq and qRT-PCR expression profiles, 10 representative genes were selected based on their expression patterns and functional annotations: LOC110094765, LOC110106494, LOC110092540, LOC110103967, LOC110110265, LOC110114476, LOC110112846, LOC110097304, LOC110104806, and LOC110113225. Total RNA was reverse-transcribed using EasyScript One-Step gDNA Removal and cDNA Synthesis SuperMix (TransGen Biotech), and qRT-PCR was performed using TranStart Tip Green qPCR SuperMix with β-Actin as the internal reference gene. The amplification program consisted of 95 °C for 30 s, followed by 40 cycles of 95 °C for 5 s and 60 °C for 34 s. Relative transcript abundance was calculated using the 2^−ΔΔCt method. Expression levels were examined at 0, 2, 4, and 6 d during chilling and at 9 d, corresponding to the first day of recovery, with three biological replicates per sampling point. Gene identities and functional annotations are provided in **Supplementary Table S6**, and the qRT-PCR profiles are shown in **Supplementary Figure S1**. To quantify concordance between the two methods, log2 fold changes at 4 d relative to 0 d were compared for the 10 genes using Pearson correlation analysis (**Supplementary Figure S2**).

### 2.7 Co-expression network analysis and identification of trait-associated modules

Weighted gene co-expression network analysis (WGCNA) was performed in R using the WGCNA package (Langfelder and Horvath, 2008). Only the 18 RNA-seq libraries from EBR-treated plants, representing six sampling time points with three biological replicates per time point, were included in the analysis. After filtering low-expression genes, 9,053 genes were retained for network construction.

Gene expression profiles based on FPKM values were integrated with chilling-related physiological traits, including malondialdehyde (MDA), soluble protein (SP), soluble sugar (SS), relative chlorophyll content (CHL), and proline content (Pro). These physiological data were obtained from the same experimental framework and sampling schedule and were originally reported by Wei et al. (2025). In the WGCNA input, trait data were aligned with the corresponding transcriptomic sampling time points. Because physiological sampling was performed independently from RNA-seq library preparation, module-trait correlations were interpreted as temporal associations rather than as evidence of sample-level causal regulation.

A soft-thresholding power of β = 18 was selected based on the scale-free topology criterion. The adjacency matrix was transformed into a topological overlap matrix (TOM), and genes were hierarchically clustered using TOM-based dissimilarity. Modules were identified using the dynamic tree-cut algorithm with a minimum module size of 30 genes, and similar modules were merged using a cut height of 0.25.

Module eigengenes were calculated and correlated with physiological traits using Pearson correlation analysis. Correlation strength, direction, and statistical significance were jointly considered to identify biologically relevant modules. Among all modules, the pink and antiquewhite4 (AW4) modules showed the most pronounced and contrasting associations with CHL, Pro, and MDA and were selected for downstream functional interpretation.

GO and KEGG enrichment analyses were performed for genes within the pink and AW4 modules to characterize their associated biological processes and metabolic pathways. Due to the absence of a fully validated module membership and network topology framework for robust hub gene prioritization, hub gene identification was not performed, and analyses were focused at the module level.

### 2.8 Cross-cultivar and developmental-stage assessment of the selected EBR treatment

Five representative *Dendrobium* cultivars were evaluated to examine whether the 2.0 mg/L EBR treatment produced comparable phenotypic responses across different cultivar backgrounds. Cultivar names were retained as recorded in the original experimental source files.

For each cultivar, plants were grouped into old, medium-sized, and small seedling classes where material was available. Because not all cultivar-by-stage combinations were available, the cross-cultivar experiment was interpreted as a descriptive assessment of phenotypic response rather than as a balanced factorial test of genotype-by-developmental-stage effects.

Three individual plants were used as biological replicates for each available treatment combination. Phenotypic assessment followed the same chilling and recovery framework described above.

### 2.9 Statistical analysis

Data were organized in Microsoft Excel 2013 and analyzed using IBM SPSS Statistics 25, Origin 2022, and GraphPad Prism 9.5. Unless otherwise stated, experiments included three biological replicates and values are presented as mean ± SD. For experiments involving multiple EBR concentrations, data were analyzed using one-way analysis of variance (ANOVA), followed by Dunnett’s multiple-comparison test using the untreated control (CK) as the reference group. Pairwise CK-versus-EBR comparisons were evaluated using independent-sample t-tests where appropriate. Differences were considered statistically significant at *p* < 0.05.

## 3 Results

### 3.1 Concentration-dependent effects of EBR on leaf senescence

A preliminary EBR concentration screen showed that the delay of leaf senescence was concentration- and stage-dependent (**Figure 1**). The response was evaluated using the proportions of 50% yellow leaves, 100% yellow leaves, and fully abscised leaves.

At the 50% yellow-leaf endpoint, all tested EBR concentrations (0.01, 0.1, 1.0, and 1.5 mg/L) showed lower values than their corresponding CK groups and were assigned to a different significance group in **Figure 1**. At the 100% yellow-leaf endpoint, no EBR concentration differed significantly from CK, whereas at the complete leaf-abscission endpoint, 1.0 mg/L EBR showed a significant reduction relative to CK. These results indicate a concentration- and stage-dependent response. Because the preliminary screen did not include 2.0 mg/L EBR, the concentration used for the transcriptomic experiment was based on the subsequent refined dose-response experiment reported by Wei et al. (2025), in which 2.0 mg/L provided the strongest overall protection among 0-2.0 mg/L treatments. Accordingly, 2.0 mg/L EBR was selected for transcriptomic analysis.

### 3.2 Physiological responses associated with the selected EBR treatment

The physiological traits used for WGCNA were obtained from the same experimental system and were reported previously by Wei et al. (2025). Specifically, MDA, soluble protein, soluble sugar, chlorophyll, and proline measurements were reused as external traits for module-trait association analysis. These measurements are not presented here as newly generated physiological results; the original data, replicate information, and statistical comparisons are available in Wei et al. (2025).

### 3.3 RNA-seq quality assessment and qRT-PCR validation

Across the 36 CK and EBR libraries, clean-read ratios ranged from approximately 99.95% to 99.98%, and Q30 values ranged from approximately 96.68% to 98.13%, indicating high sequencing quality after filtering (**Supplementary Table S1**). However, genome-alignment rates for most libraries were relatively low, ranging from approximately 17.75% to 21.17%. Notably, CK13d_1, CK13d_2, and CK13d_3 exhibited exceptionally low mapping rates of approximately 0.06%, 0.08%, and 0.08%, respectively. These three libraries also showed elevated GC contents (approximately 53.72–54.05%) compared with the approximately 45–46% observed for most other libraries, suggesting a potential sample-identity, contamination, or reference-compatibility issue. Therefore, CK-versus-EBR comparisons at 13 d were treated with caution and were not used for strong biological inference pending further verification of these libraries.

To assess concordance between RNA-seq and qRT-PCR expression profiles, 10 representative genes were selected for validation: LOC110092540 (serine--glyoxylate aminotransferase), LOC110104806 (fructose-bisphosphate aldolase 1, chloroplastic), LOC110110265 (glycine dehydrogenase [decarboxylating], mitochondrial), LOC110097304 (probable glutathione S-transferase parA), LOC110106494 (chlorophyll a-b binding protein 3, chloroplastic), LOC110114476 (serine hydroxymethyltransferase, mitochondrial), LOC110103967 (pyruvate, phosphate dikinase, chloroplastic), LOC110112846 (glycerate dehydrogenase), LOC110094765 (phosphoenolpyruvate carboxylase 2), and LOC110113225 (GTP-binding protein SAR1A-like) (**Supplementary Table S6**). The qRT-PCR time-course profiles are shown in **Supplementary Figure S1**. Expression changes at 4 d relative to 0 d were compared for the 10 validated genes. Pearson correlation analysis revealed a strong positive relationship between the two methods (r = 0.950, P < 0.001; **Supplementary Figure S2**), indicating good concordance between RNA-seq and qRT-PCR expression changes for the selected genes.

### 3.4 Differential expression responses to EBR during chilling and recovery

Differential-expression analysis revealed time-dependent transcriptional responses to EBR. At 4 d of chilling, 1,391 DEGs were detected in EBR versus CK, including 489 upregulated and 902 downregulated genes. Because the three CK13d libraries showed extreme mapping failure, the 13-d CK-versus-EBR comparison was not used for biological interpretation. Global GO and KEGG outputs from the archived analysis are retained in **Figures 2**, **3** as descriptive overviews of functional classification and nominal enrichment patterns. Because complete multiple-testing-adjusted statistics could not be reconstructed for these global comparisons, individual terms or pathways in **Figures 2** and **3** are not interpreted as statistically significant in the revised manuscript. Quantitative functional inference is instead based on the fully documented module-specific enrichment analyses in Section 3.6 and **Supplementary Tables S2**–**S5**.

**Figure 2:**
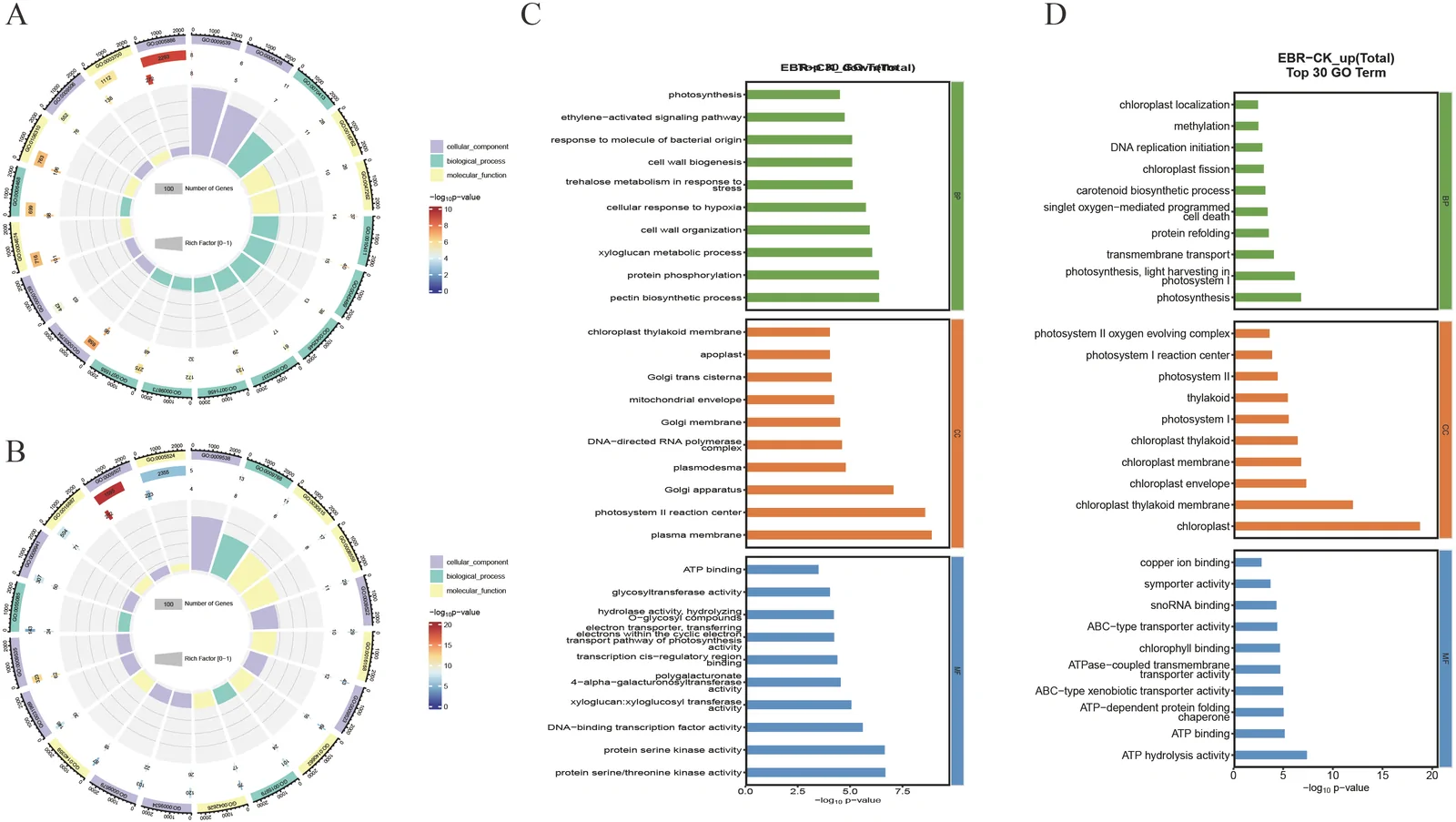
Global GO functional classification and exploratory enrichment profiles of EBR-responsive genes during chilling. **(A, B)** summarize GO functional classifications, whereas **(C, D)** show representative GO terms among downregulated and upregulated genes, respectively. The enrichment panels are retained as descriptive overviews of the archived global analysis; because complete multiple-testing-adjusted statistics could not be reconstructed for these global comparisons, the displayed terms are not used to define statistically significant GO enrichment in the revised manuscript. Quantitative inference is based on the module-specific analyses in **Supplementary Tables S2**, **S3**.

**Figure 3:**
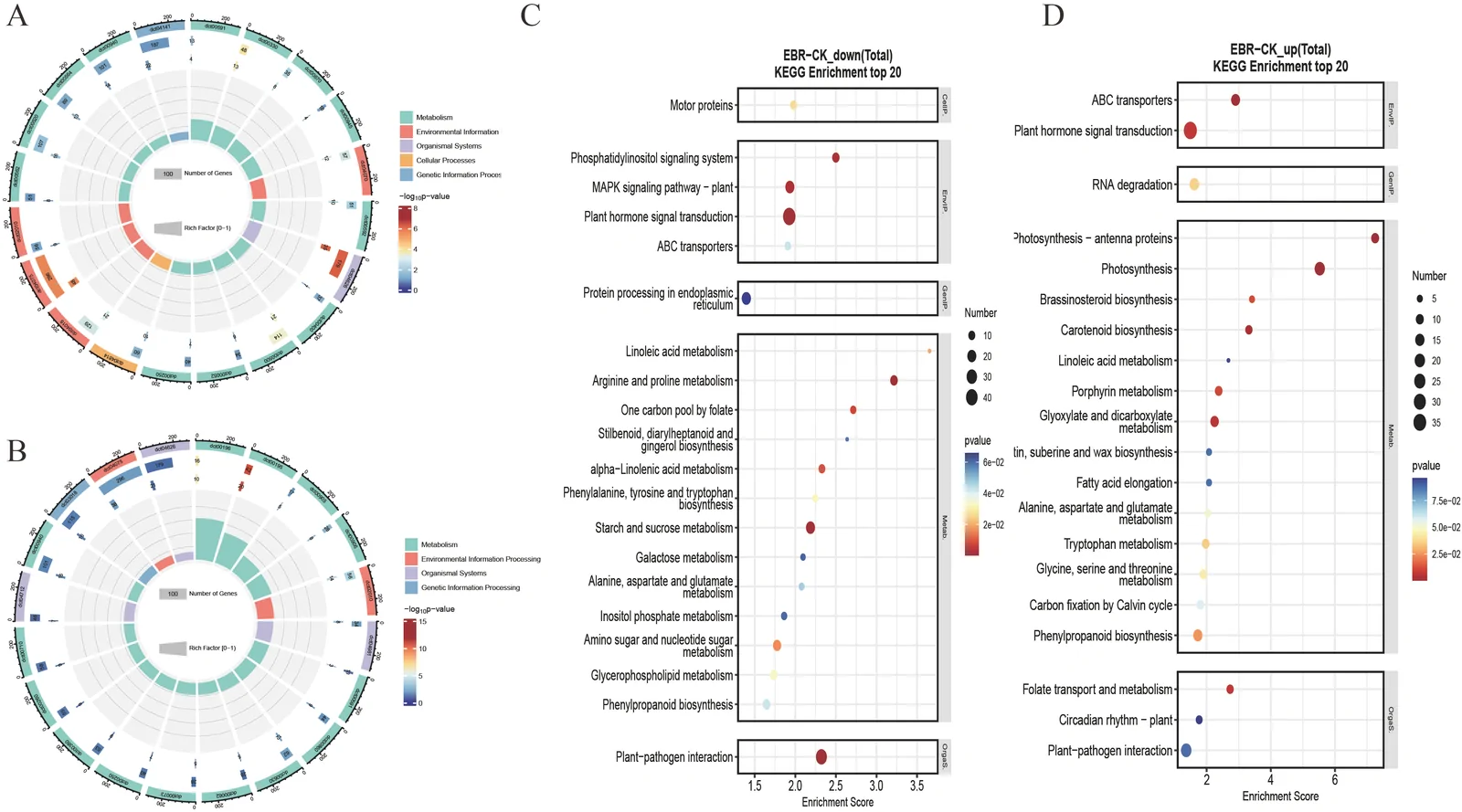
Global KEGG pathway classification and exploratory enrichment profiles of EBR-responsive genes during chilling. **(A, B)** summarize KEGG pathway classifications, whereas **(C, D)** display representative pathways among downregulated and upregulated genes, respectively. These plots are retained as descriptive visualizations of the archived global analysis and are not interpreted as multiple-testing-corrected significant pathway sets. Quantitative pathway-level inference is restricted to the module-specific results in **Supplementary Tables S4**, **S5**.

### 3.5 Co-expression network construction and trait-associated modules

WGCNA identified multiple co-expression modules from 9,053 expressed genes across 18 EBR-treated samples. Hierarchical clustering based on TOM dissimilarity revealed distinct gene modules with coordinated expression patterns across developmental time points.

Module-trait correlation analysis identified pronounced and contrasting associations between the pink and AW4 modules and several physiological traits (**Figure 4B**). The pink module was positively correlated with MDA (r = 0.83) and proline (r = 0.95), but negatively correlated with chlorophyll content (r = -0.83). In contrast, the AW4 module was negatively correlated with MDA (r = -0.70) and proline (r = -0.94), while showing a strong positive correlation with chlorophyll content (r = 0.94). Associations with soluble protein and soluble sugar were weaker or module dependent.

**Figure 4:**
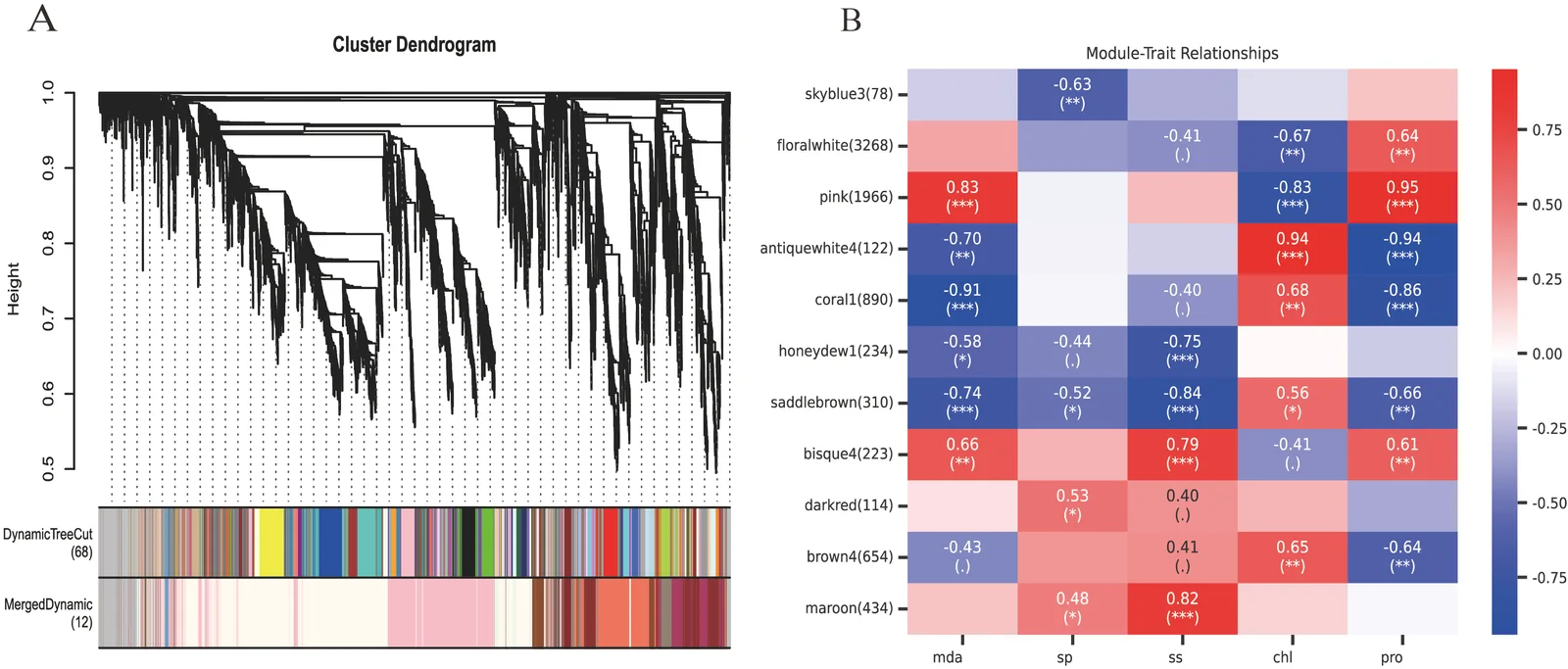
Identification of trait-associated co-expression modules in EBR-treated Dendrobium using WGCNA. **(A)** Gene dendrogram based on TOM dissimilarity with dynamic-tree-cut and merged module colors. **(B)** Module-trait relationship heatmap for MDA, soluble protein (SP), soluble sugar (SS), chlorophyll (CHL), and proline (Pro). Cell values indicate Pearson correlation coefficients; symbols denote the significance levels shown in the original WGCNA output. * indicates P < 0.05, ** indicates P < 0.01, and *** indicates P < 0.001.

These contrasting temporal associations indicate that the pink and AW4 modules capture distinct co-expression patterns related to physiological variation during EBR treatment. Because both module eigengenes and physiological traits vary across the same time course, these relationships are interpreted as associations rather than direct causal regulation. The two modules were selected for module-specific functional enrichment analysis.

Module-specific enrichment did not support simple labels such as a ‘photosynthesis module’ or ‘antioxidant module.’ Instead, the pink module showed significant enrichment for central carbon and energy metabolism, lipid turnover, peroxisomal processes, autophagy, and cellular transport/homeostasis, whereas significant AW4 GO terms were dominated by transmembrane, mitochondrial, organic-acid, and ion transport. No AW4 KEGG pathway remained significant after multiple-testing correction.

### 3.6 Module-specific functional enrichment

Module-specific enrichment showed that the pink module should not be interpreted as a direct photosynthesis/proline-biosynthesis module. Of the 1,966 pink-module genes, 1,626 were represented in the GO annotation set. Seventeen GO terms remained significant at q < 0.05 (**Supplementary Table S2**), including endoplasmic reticulum, plasma membrane, protein transport, plant-type vacuole, autophagosome membrane, Golgi membrane/apparatus, mitochondrial electron transport from succinate to ubiquinone, the tricarboxylic acid cycle, NAD+ binding, ubiquitin-protein transferase activity, antiporter activity, protein dephosphorylation, and acetyl-CoA biosynthesis from pyruvate. KEGG analysis included 556 annotated pink-module genes and identified 11 significant pathways (q < 0.05; **Supplementary Table S4**): pantothenate and CoA biosynthesis (15 genes, enrichment score = 4.061, q = 6.92 × 10^-5), citrate cycle/TCA cycle (16 genes, 3.052, q = 0.001396), glycerolipid metabolism (19 genes, 2.246, q = 0.016201), peroxisome (21 genes, 2.123, q = 0.016201), fatty acid degradation (13 genes, 2.537, q = 0.025756), autophagy-other (11 genes, 2.564, q = 0.045863), stilbenoid/diarylheptanoid/gingerol biosynthesis (7 genes, 3.264, q = 0.045863), pyruvate metabolism (20 genes, 1.886, q = 0.045863), valine/leucine/isoleucine degradation (11 genes, 2.429, q = 0.045863), ether lipid metabolism (8 genes, 2.919, q = 0.045863), and cysteine and methionine metabolism (20 genes, 1.844, q = 0.049108). Arginine and proline metabolism, glutathione metabolism, and oxidative phosphorylation were not significant after multiple-testing correction (**Supplementary Table S4**).

For AW4, 102 of the 122 module genes were represented in the GO annotation set. Eighteen GO terms remained significant at q < 0.05 (**Supplementary Table S3**), with significant terms concentrated in transmembrane/metabolite transport and related transporter activities, including thiosulfate, sulfate, succinate, oxaloacetate, malate, and dicarboxylic-acid transport, as well as mitochondrial transport. Several of the most highly enriched terms were supported by the same small set of genes and should therefore not be treated as independent mechanistic signals. KEGG analysis included 35 AW4 genes; nine pathways had raw P < 0.05, but none remained significant after multiple-testing correction (all q = 0.159573; **Supplementary Table S5**). These nominal pathways included starch and sucrose metabolism, plant MAPK signaling, steroid biosynthesis, glycolysis/gluconeogenesis, and the pentose phosphate pathway (**Supplementary Table S5**).

### 3.7 Cross-cultivar and developmental-stage responses to the selected EBR treatment

Qualitative time-series observations indicated that EBR-treated plants generally retained greener leaves and delayed visible senescence relative to CK in several cultivar-by-stage combinations (**Figures 5A, B**). The magnitude and timing of the visible response varied among cultivars and developmental stages.

**Figure 5:**
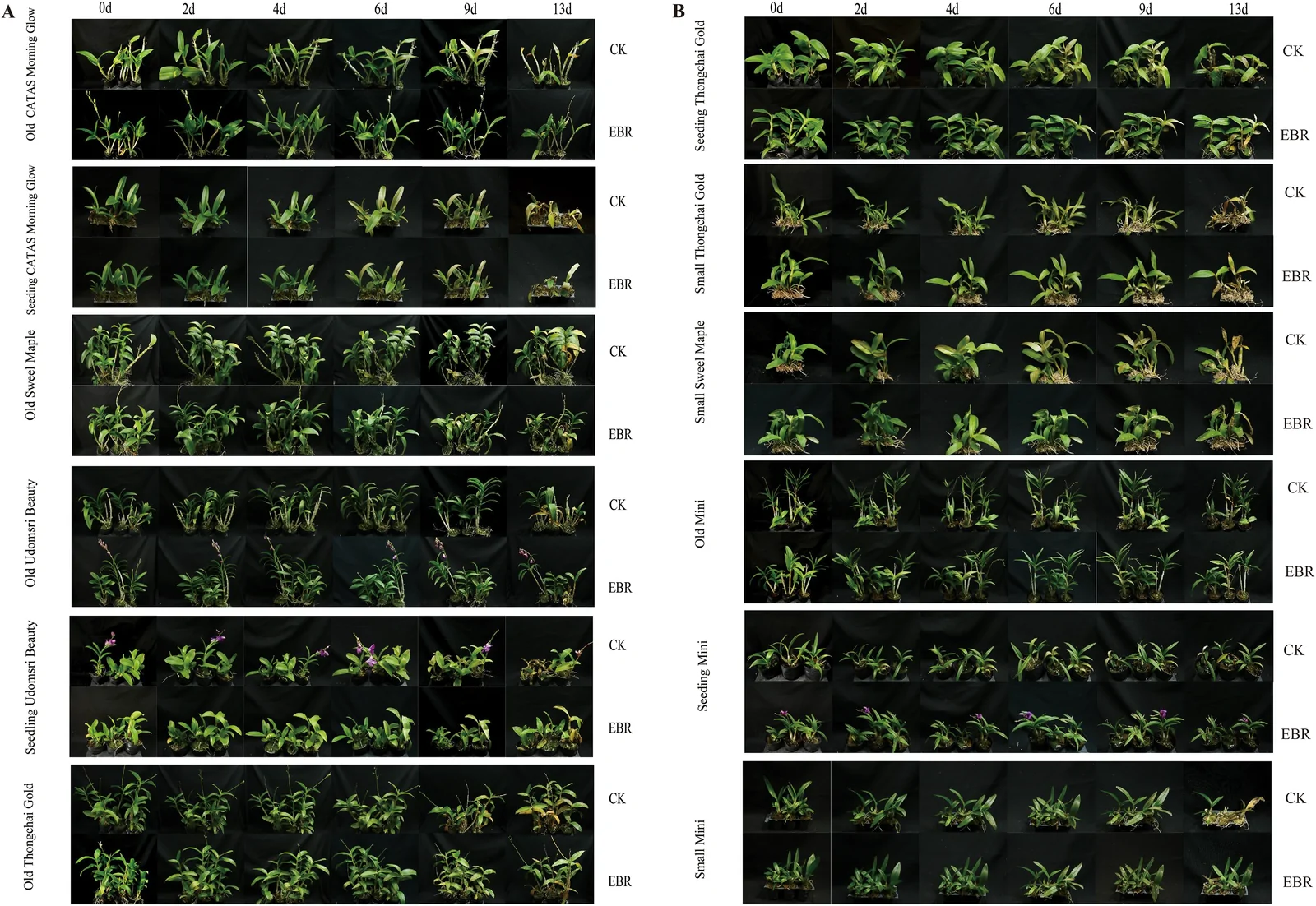
**(A)** Phenotypic responses of *Dendrobium* cultivar-by-developmental-stage combinations to chilling under CK and EBR treatments. **(B)** Phenotypic responses of additional *Dendrobium* cultivar-by-developmental-stage combinations to chilling under CK and EBR treatments.

Some cultivar-by-stage combinations also showed differences in plant compactness or flowering status during recovery; because these traits were assessed visually rather than by a balanced quantitative experiment, they are presented as descriptive observations rather than as evidence that EBR directly promotes cell proliferation or flowering.

The variation among cultivars is therefore described as cultivar-dependent variation in EBR responsiveness. The present experiment did not include comparative transcriptomic or genotypic analyses across cultivars and cannot resolve the genetic basis of these differences.

These observations suggest that EBR-associated visible responses occur across multiple *Dendrobium* backgrounds, while also indicating that cultivar- and developmental-stage-specific optimization may be required.

## 4 Discussion

### 4.1 Exogenous EBR alleviates chilling-associated senescence and physiological injury

Exogenous EBR substantially alleviated chilling-associated leaf yellowing and abscission in *Phalaenopsis-type Dendrobium*. Together with the previously characterized physiological responses, including better maintenance of chlorophyll and osmolyte-related traits and lower MDA accumulation and electrolyte leakage under the selected EBR treatment, these observations indicate that EBR helps preserve cellular homeostasis during chilling. However, these physiological changes likely arise from multiple interacting processes rather than from a single downstream mechanism.

The maintenance of leaf greenness under EBR treatment is broadly consistent with the protective effects of brassinosteroids reported in other plant species. In rice, application of plant growth regulators including BRs has been associated with improved photosynthetic performance under environmental stress (Fahad et al., 2016). Similarly, BR-mediated regulation of photosynthesis-related processes has been reported in maize, where maintenance of light-harvesting functions contributes to stress acclimation (Li et al., 2025a). These findings provide a useful comparative framework for interpreting the delayed chlorophyll loss observed in Dendrobium. Nevertheless, because chlorophyll-degrading enzymes and photosynthetic parameters were not directly examined in the present transcriptomic experiment, the maintenance of leaf greenness should be interpreted as a physiological outcome rather than direct evidence for regulation of a specific chlorophyll-degradation pathway.

Leaf abscission is a highly coordinated developmental process involving hormonal signaling and cell-wall remodeling. Auxin and ethylene are central regulators of abscission-zone activation, with ethylene generally promoting abscission and auxin maintaining tissue resistance to ethylene signaling (Zhang et al., 2009). Previous studies have also shown that brassinosteroids interact with other phytohormone pathways and may influence developmental and stress-related processes involving auxin and ethylene (Mao et al., 2017). In litchi, BR-related regulation has been associated with auxin signaling, ethylene-related responses, and cell-wall modification during organ abscission (Ma et al., 2021). The delayed leaf shedding observed in EBR-treated *Dendrobium* is therefore compatible with the possibility that EBR influences hormone-dependent abscission processes. However, endogenous auxin and ethylene levels, abscission-zone gene expression, and cell-wall hydrolase activities were not measured here. Thus, regulation of auxin/ethylene homeostasis remains a testable hypothesis rather than a demonstrated mechanism in *Dendrobium*.

The concentration-dependent response further indicates that EBR activity does not follow a simple linear dose relationship. Different EBR concentrations affected early yellowing and later leaf abscission to different extents, whereas 2.0 mg/L provided the strongest overall protection in the subsequent refined experiment. Such variation emphasizes that the physiological outcome of BR application may depend on concentration, developmental stage, and the specific response endpoint. Therefore, additional controlled studies will be required before stage-specific or production-scale application regimes can be recommended.

### 4.2 The pink module is associated with stress-related physiological variation and broader metabolic homeostasis

WGCNA revealed that the pink module was positively associated with MDA and proline but negatively associated with chlorophyll. Because WGCNA identifies coordinated temporal expression patterns rather than direct regulatory relationships, these correlations should not be interpreted as evidence that pink-module genes directly increase MDA or proline or decrease chlorophyll. Instead, they indicate that the module eigengene covaries with a physiological state characterized by greater stress-associated metabolite accumulation and reduced chlorophyll during the time course.

The significant GO and KEGG terms in the pink module were mainly associated with central carbon and energy metabolism, including the TCA cycle, pyruvate metabolism, and pantothenate and CoA biosynthesis, together with lipid turnover, peroxisomal processes, autophagy, and intracellular protein and organelle transport. Notably, canonical photosynthesis and arginine/proline metabolism were not significantly enriched after multiple-testing correction. Thus, the opposing associations of the pink module with MDA/proline and chlorophyll most plausibly reflect broader metabolic and cellular-state changes rather than direct overrepresentation of photosynthesis or proline-biosynthesis pathways.

This interpretation is compatible with observations in other species. Brassinosteroid-mediated enhancement of abiotic-stress tolerance has frequently been associated with improved osmotic adjustment and maintenance of metabolic activity, including proline-related responses in tomato and maize (Sharma et al., 2019). Similarly, coordinated regulation of energy metabolism, stress-responsive transcription, and osmotic adjustment has been reported in wheat under abiotic stress (Lv et al., 2020). These studies suggest that maintenance of chlorophyll and accumulation of compatible solutes can occur as part of a wider metabolic adjustment rather than through isolated regulation of individual biosynthetic pathways.

Accordingly, the pink module may represent a stress-associated metabolic program whose activity increases as MDA and proline rise and chlorophyll declines. Its enrichment for TCA-cycle and pyruvate metabolism, lipid turnover, peroxisomal and autophagic processes, and cellular transport suggests coordinated metabolic remodeling and homeostasis rather than a dedicated photosynthesis or proline-biosynthesis pathway. Further gene-level analyses will be required to determine which components of this module contribute directly to these physiological responses.

### 4.3 The AW4 module is associated with MDA/chlorophyll variation and membrane-related transport

The AW4 module showed a strong negative association with MDA content. MDA is a major product of membrane lipid peroxidation and is widely used as an indicator of oxidative membrane injury rather than as a metabolic substrate itself (Ayala et al., 2014; Khyade et al., 2019). Therefore, the inverse relationship between AW4 expression and MDA suggests that the temporal behavior of this module is associated with variation in membrane-injury status during chilling and recovery.

Such an association is broadly consistent with reports of BR-mediated membrane protection in other plant species. For example, exogenous brassinosteroids improved chilling tolerance in cucumber through enhanced antioxidant capacity and protection of cellular membranes (Fariduddin et al., 2011). However, the enrichment profile of AW4 in the present study does not support describing this module as a direct antioxidant module. Significant AW4 GO terms were primarily related to transmembrane transport, mitochondrial transport, organic-acid transport, sulfate and ion transport, and related transporter activities. Moreover, none of the AW4 KEGG pathways remained significant after multiple-testing correction.

Together with its positive association with chlorophyll and negative association with proline, the inverse relationship between AW4 and MDA suggests that AW4 expression covaries with a comparatively less-injured physiological state. Its transport-dominated enrichment may reflect broader maintenance of membrane and organelle homeostasis rather than direct enrichment of classical ROS-scavenging pathways. Low temperature alters membrane properties, mitochondrial activity, ion balance, and metabolite exchange among cellular compartments; regulation of transport-related processes may therefore indirectly contribute to cellular adjustment during chilling.

The pink and AW4 modules therefore show opposing trait-association patterns: pink tracks a state with higher MDA and proline and lower chlorophyll, whereas AW4 tracks lower MDA and proline and higher chlorophyll. Their functional profiles also differ, with pink enriched for broad metabolic and homeostatic processes and AW4 enriched primarily for transport-related processes. Nevertheless, these relationships remain correlative. Because verified module-membership, gene-significance, and intramodular-connectivity outputs were not available for the archived pink and AW4 analyses, individual hub genes were not nominated in the revised manuscript. Future reconstruction of gene-level network topology and functional validation will be necessary to determine the regulatory components underlying these module-level associations.

### 4.4 Cultivar- and developmental-stage-dependent EBR responsiveness in comparison with other species

The phenotypic observations across multiple *Dendrobium* cultivars indicate that EBR-associated responses are not restricted to a single cultivar background. EBR-treated plants generally maintained greener leaves and delayed visible senescence, although the magnitude and timing of the response varied among cultivar-by-developmental-stage combinations. Some plants also exhibited differences in compactness or flowering status during recovery. Because these traits were evaluated primarily by visual observation rather than through a fully balanced quantitative experiment, they should be interpreted descriptively rather than as direct evidence that EBR promotes cell proliferation or flowering.

The general anti-senescence phenotype observed in *Dendrobium* is consistent with reports of BR-mediated maintenance of plant growth and physiological activity in other species. Brassinosteroids have been associated with improved photosynthetic performance and reduced stress-induced deterioration in several crops, including soybean and other species exposed to environmental stress (Barros Junior et al., 2021). Exogenous BRs have also been reported to enhance physiological performance and stress tolerance in cucumber and other plants (Anwar et al., 2018; Guedes et al., 2021; Lu et al., 2025). These observations support a broadly conserved role of BRs in maintaining plant physiological activity under adverse conditions, while the specific responses in *Dendrobium* remain dependent on cultivar and developmental stage.

Brassinosteroids are also involved in reproductive development in several plant species. Effects on flowering-related traits, pollen viability, and reproductive development have been reported in tulip and pomegranate (Akçal et al., 2016; Gökbayrak and Engin, 2018). The differences in flowering status observed among some EBR-treated *Dendrobium* plants are therefore compatible with known developmental functions of BRs. However, flowering time, flower number, floral organ development, and flowering-related genes were not quantitatively evaluated in the present study. Consequently, the current observations do not establish a direct promotive effect of EBR on flowering in *Dendrobium*.

Developmental stage may also influence EBR responsiveness. Previous studies have associated brassinosteroid signaling with stress adaptation, senescence, and maintenance of physiological activity under adverse conditions (Wei et al., 2015; Nie et al., 2024; Wei et al., 2024). The relatively pronounced anti-senescence response observed in some older *Dendrobium* plants may therefore reflect differences in developmental status and stress sensitivity. BR-mediated improvement of antioxidant and physiological performance has likewise been reported under different environmental stresses (Guedes et al., 2021). Nevertheless, because the cultivar-by-stage combinations in the present study were not fully balanced, age-dependent responses should be regarded as descriptive observations requiring further controlled validation.

The variation among cultivars is also consistent with the general observation that plant genotypes differ in their responsiveness to exogenous growth regulators. Genotype-dependent differences in BR-mediated growth and stress responses have been reported in crops such as canola and soybean (Raza et al., 2021; de Brito et al., 2025). Such variation may potentially involve endogenous BR homeostasis, receptor activity, downstream signaling, or differences in general stress-response capacity. In the present study, however, comparative transcriptomic analysis, endogenous BR quantification, and genotyping were not performed across cultivars. Therefore, the observed differences are more appropriately described as cultivar-dependent EBR responsiveness rather than as evidence for a defined genetic basis.

### 4.5 Study limitations and implications

Several limitations should be considered when interpreting the present study. First, reference-genome mapping rates for most libraries were only approximately 17.75-21.17%, which is low and may reflect, at least in part, reference-genome compatibility with the *Phalaenopsis-type Dendrobium* hybrid material. In addition, the three CK13d libraries showed extreme mapping failure (approximately 0.06-0.08%) together with elevated GC content (approximately 53.72-54.05%). Conclusions that depend on CK13d comparisons were therefore not retained for biological interpretation.

Second, endogenous BR concentrations were not measured. The previous interpretation of BR-related enrichment as a positive-feedback loop was therefore removed, and the present manuscript does not infer increased endogenous BR production from transcriptomic data alone. Third, verified ImConn/MM/GS outputs for the pink and AW4 modules were not available in the archived analysis package; accordingly, no hub genes or hub-gene networks were nominated in the revised manuscript. These module-level associations require future computational and experimental validation at the gene level.

Finally, the physiological dataset used to support EBR concentration selection and to provide WGCNA traits was published previously (Wei et al., 2025). The present manuscript therefore cites those measurements as previously published supporting data and does not reproduce the earlier physiological figures as newly generated results.

Despite these limitations, the integrated physiological, differential-expression, and co-expression analyses consistently indicate that EBR treatment is associated with coordinated metabolic, membrane-homeostasis, and stress-response adjustments during chilling and recovery in *Phalaenopsis-type Dendrobium*.

## Data Availability

The raw sequence data reported in this paper have been deposited in the Genome Sequence Archive (Genomics, Proteomics & Bioinformatics 2025) in National Genomics Data Center (Nucleic Acids Research 2025), China National Center for Bioinformation / Beijing Institute of Genomics, Chinese Academy of Sciences (GSA: CRA049891), and are publicly accessible at https://ngdc.cncb.ac.cn/gsa/.
