# Supplementary material for: Exogenous 24-epibrassinolide enhances chilling tolerance in Phalaenopsis-type Dendrobium through coordinated physiological and transcriptomic responses
Source: Front Plant Sci. 2026 Sep 18;17:1912617. doi: 10.3389/fpls.2026.1912617 (PMC13632489; doi:10.3389/fpls.2026.1912617)
Supplement: Supplementary file 1 [file SupplementaryFile1.docx]

**Supplementary Tables**

*Exogenous 24-Epibrassinolide Enhances Chilling Tolerance in Phalaenopsis-Type Dendrobium through Coordinated Physiological and Transcriptomic Responses*

Supplementary Table S1. RNA-seq sequencing quality and reference-genome mapping statistics for CK and EBR libraries.

The transcriptome dataset used for CK–EBR comparisons comprised 36 libraries (2 treatments × 6 time points × 3 biological replicates). Percentages are shown as %. Mapping statistics are reported exactly as provided by the original mapping output.

| **Sample** | **Treatment** | **Time (d)** | **Replicate** | **Raw reads** | **Clean reads** | **Clean ratio (%)** | **Q20 (%)** | **Q30 (%)** | **GC (%)** | **Mapped reads** | **Mapping rate (%)** | **Unique mapped reads** | **Paired mapped reads** | **Single mapped reads** | **Self-and-mate reads** | **Mapped to different chromosomes** |
| --- | --- | --- | --- | --- | --- | --- | --- | --- | --- | --- | --- | --- | --- | --- | --- | --- |
| Ck_0d_1 | CK | 0 | 1 | 63,430,328 | 63,413,558 | 99.97 | 99.27 | 97.47 | 45.86 | 13,275,169 | 20.93 | 12,766,058 | 7,455,924 | 5,461,815 | 7,813,354 | 30,422 |
| Ck_0d_2 | CK | 0 | 2 | 78,399,058 | 78,377,780 | 99.97 | 99.41 | 97.92 | 45.47 | 15,759,767 | 20.11 | 15,123,242 | 9,019,164 | 6,290,147 | 9,469,620 | 36,682 |
| Ck_0d_3 | CK | 0 | 3 | 74,058,336 | 74,038,456 | 99.97 | 99.40 | 97.88 | 45.44 | 14,944,830 | 20.19 | 14,332,134 | 8,304,364 | 6,212,492 | 8,732,338 | 35,140 |
| Ck_2d_1 | CK | 2 | 1 | 77,554,732 | 77,531,454 | 99.97 | 99.37 | 97.77 | 45.39 | 14,527,937 | 18.74 | 13,915,660 | 7,824,742 | 6,261,821 | 8,266,116 | 42,014 |
| Ck_2d_2 | CK | 2 | 2 | 66,251,258 | 66,232,666 | 99.97 | 99.38 | 97.83 | 45.34 | 12,388,300 | 18.70 | 11,861,565 | 6,635,042 | 5,373,612 | 7,014,688 | 35,784 |
| Ck_2d_3 | CK | 2 | 3 | 65,184,788 | 65,166,108 | 99.97 | 99.41 | 97.92 | 45.35 | 12,099,319 | 18.57 | 11,612,405 | 6,580,102 | 5,170,769 | 6,928,550 | 34,568 |
| Ck_4d_1 | CK | 4 | 1 | 47,963,466 | 47,952,080 | 99.98 | 98.93 | 96.68 | 45.20 | 9,508,903 | 19.83 | 8,566,744 | 5,055,748 | 3,641,107 | 5,867,796 | 22,492 |
| Ck_4d_2 | CK | 4 | 2 | 64,939,022 | 64,921,644 | 99.97 | 99.42 | 97.95 | 45.42 | 13,048,985 | 20.10 | 11,760,414 | 6,783,578 | 5,150,115 | 7,898,870 | 32,490 |
| Ck_4d_3 | CK | 4 | 3 | 63,176,794 | 63,160,126 | 99.97 | 99.32 | 97.59 | 45.36 | 12,670,174 | 20.06 | 11,431,224 | 6,545,866 | 5,056,744 | 7,613,430 | 32,434 |
| Ck_6d_1 | CK | 6 | 1 | 70,586,768 | 70,562,312 | 99.97 | 99.48 | 98.13 | 45.02 | 14,576,592 | 20.66 | 11,137,359 | 6,703,894 | 4,646,732 | 9,929,860 | 32,702 |
| Ck_6d_2 | CK | 6 | 2 | 55,540,448 | 55,522,004 | 99.97 | 99.41 | 97.93 | 44.99 | 11,448,410 | 20.62 | 8,762,202 | 5,195,148 | 3,731,516 | 7,716,894 | 26,670 |
| Ck_6d_3 | CK | 6 | 3 | 58,264,146 | 58,244,830 | 99.97 | 99.35 | 97.75 | 45.05 | 12,009,755 | 20.62 | 9,189,121 | 5,461,538 | 3,898,125 | 8,111,630 | 27,344 |
| Ck_9d_1 | CK | 9 | 1 | 76,483,366 | 76,460,728 | 99.97 | 99.41 | 97.92 | 46.07 | 14,659,117 | 19.17 | 13,983,514 | 7,999,470 | 6,151,631 | 8,507,486 | 38,462 |
| Ck_9d_2 | CK | 9 | 2 | 69,644,962 | 69,624,050 | 99.97 | 99.41 | 97.92 | 45.97 | 13,388,048 | 19.23 | 12,829,110 | 7,339,542 | 5,636,460 | 7,751,588 | 35,538 |
| Ck_9d_3 | CK | 9 | 3 | 65,447,502 | 65,428,482 | 99.97 | 99.41 | 97.90 | 45.99 | 12,575,246 | 19.22 | 12,002,717 | 6,888,954 | 5,255,172 | 7,320,074 | 33,668 |
| Ck_13d_1 | CK | 13 | 1 | 50,521,382 | 50,495,852 | 99.95 | 99.21 | 97.28 | 54.05 | 28,465 | 0.06 | 16,290 | 5,644 | 14,587 | 13,878 | 6 |
| Ck_13d_2 | CK | 13 | 2 | 60,502,944 | 60,472,770 | 99.95 | 99.29 | 97.55 | 53.72 | 47,951 | 0.08 | 26,365 | 9,414 | 23,187 | 24,764 | 8 |
| Ck_13d_3 | CK | 13 | 3 | 67,557,454 | 67,523,940 | 99.95 | 99.29 | 97.53 | 53.74 | 56,710 | 0.08 | 30,441 | 10,366 | 27,614 | 29,096 | 8 |
| EBR_0d_1 | EBR | 0 | 1 | 71,545,006 | 71,525,094 | 99.97 | 99.38 | 97.81 | 46.14 | 14,991,023 | 20.96 | 14,304,039 | 8,559,090 | 5,942,599 | 9,048,424 | 32,910 |
| EBR_0d_2 | EBR | 0 | 2 | 65,925,354 | 65,907,932 | 99.97 | 99.40 | 97.90 | 46.19 | 13,954,028 | 21.17 | 13,319,547 | 7,756,632 | 5,749,968 | 8,204,060 | 32,426 |
| EBR_0d_3 | EBR | 0 | 3 | 68,928,700 | 68,910,244 | 99.97 | 99.43 | 97.99 | 46.13 | 14,571,870 | 21.15 | 13,902,755 | 8,108,862 | 5,992,718 | 8,579,152 | 33,594 |
| EBR_2d_1 | EBR | 2 | 1 | 64,177,928 | 64,161,942 | 99.98 | 99.33 | 97.66 | 45.21 | 11,775,952 | 18.35 | 11,326,180 | 6,517,660 | 4,936,878 | 6,839,074 | 34,788 |
| EBR_2d_2 | EBR | 2 | 2 | 61,144,264 | 61,127,998 | 99.97 | 99.39 | 97.86 | 45.21 | 11,224,196 | 18.36 | 10,795,450 | 6,147,720 | 4,770,560 | 6,453,636 | 33,752 |
| EBR_2d_3 | EBR | 2 | 3 | 68,454,608 | 68,437,384 | 99.97 | 99.42 | 97.95 | 45.16 | 12,595,233 | 18.40 | 12,105,424 | 7,016,878 | 5,225,571 | 7,369,662 | 36,526 |
| EBR_4d_1 | EBR | 4 | 1 | 64,891,642 | 64,874,254 | 99.97 | 99.34 | 97.70 | 45.19 | 11,585,004 | 17.86 | 11,053,475 | 6,203,306 | 5,009,576 | 6,575,428 | 33,236 |
| EBR_4d_2 | EBR | 4 | 2 | 64,320,992 | 64,303,396 | 99.97 | 99.45 | 98.06 | 45.35 | 11,415,618 | 17.75 | 10,902,367 | 6,661,706 | 4,371,676 | 7,043,942 | 28,726 |
| EBR_4d_3 | EBR | 4 | 3 | 64,126,000 | 64,108,730 | 99.97 | 99.36 | 97.78 | 45.31 | 11,475,871 | 17.90 | 10,944,634 | 6,241,814 | 4,857,639 | 6,618,232 | 31,858 |
| EBR_6d_1 | EBR | 6 | 1 | 46,833,812 | 46,821,294 | 99.97 | 99.41 | 97.91 | 45.34 | 8,767,819 | 18.73 | 8,126,829 | 4,668,118 | 3,593,645 | 5,174,174 | 24,378 |
| EBR_6d_2 | EBR | 6 | 2 | 57,865,374 | 57,849,344 | 99.97 | 99.42 | 97.96 | 45.30 | 10,841,260 | 18.74 | 10,049,541 | 5,839,566 | 4,368,440 | 6,472,820 | 29,506 |
| EBR_6d_3 | EBR | 6 | 3 | 62,464,364 | 62,447,926 | 99.97 | 99.36 | 97.74 | 45.26 | 11,730,372 | 18.78 | 10,889,582 | 6,187,676 | 4,875,618 | 6,854,754 | 32,542 |
| EBR_9d_1 | EBR | 9 | 1 | 66,597,798 | 66,578,646 | 99.97 | 99.46 | 98.09 | 46.25 | 13,164,492 | 19.77 | 12,485,756 | 7,191,886 | 5,439,644 | 7,724,848 | 36,528 |
| EBR_9d_2 | EBR | 9 | 2 | 77,219,276 | 77,197,594 | 99.97 | 99.38 | 97.81 | 46.09 | 15,314,774 | 19.84 | 14,566,408 | 8,430,374 | 6,306,598 | 9,008,176 | 41,630 |
| EBR_9d_3 | EBR | 9 | 3 | 72,150,316 | 72,129,826 | 99.97 | 99.37 | 97.78 | 46.18 | 14,364,887 | 19.92 | 13,646,598 | 7,795,196 | 6,012,213 | 8,352,674 | 40,032 |
| EBR_13d_1 | EBR | 13 | 1 | 67,348,646 | 67,330,018 | 99.97 | 99.46 | 98.08 | 45.05 | 13,996,634 | 20.79 | 13,478,467 | 7,785,816 | 5,842,856 | 8,153,778 | 36,710 |
| EBR_13d_2 | EBR | 13 | 2 | 59,502,682 | 59,486,000 | 99.97 | 99.42 | 97.93 | 45.12 | 12,332,349 | 20.73 | 11,881,152 | 6,856,422 | 5,155,061 | 7,177,288 | 32,480 |
| EBR_13d_3 | EBR | 13 | 3 | 64,638,372 | 64,620,650 | 99.97 | 99.40 | 97.88 | 45.10 | 13,464,927 | 20.84 | 12,959,388 | 7,353,698 | 5,753,575 | 7,711,352 | 36,008 |

Supplementary Table S2. Significant GO enrichment results for the pink WGCNA module.

Only terms with q < 0.05 are included. The pink enrichment profile is dominated by membrane/organelle-associated processes, intracellular protein transport, mitochondrial energy metabolism and protein homeostasis.

| **GO ID** | **Term** | **Category** | **List hits** | **List total** | **Background hits** | **Background total** | **P value** | **q value** | **Enrichment score** |
| --- | --- | --- | --- | --- | --- | --- | --- | --- | --- |
| GO:0005783 | endoplasmic reticulum | cellular_component | 103 | 1,626 | 613 | 16,770 | 1.653e-08 | 2.792e-05 | 1.733 |
| GO:0005886 | plasma membrane | cellular_component | 293 | 1,626 | 2,293 | 16,770 | 1.285e-07 | 1.085e-04 | 1.318 |
| GO:0015031 | protein transport | biological_process | 55 | 1,626 | 279 | 16,770 | 2.445e-07 | 1.376e-04 | 2.033 |
| GO:0000325 | plant-type vacuole | cellular_component | 64 | 1,626 | 363 | 16,770 | 1.680e-06 | 7.093e-04 | 1.818 |
| GO:0000421 | autophagosome membrane | cellular_component | 8 | 1,626 | 13 | 16,770 | 6.327e-06 | 0.002137 | 6.347 |
| GO:0000139 | Golgi membrane | cellular_component | 70 | 1,626 | 442 | 16,770 | 2.586e-05 | 0.007279 | 1.633 |
| GO:0006121 | mitochondrial electron transport, succinate to ubiquinone | biological_process | 5 | 1,626 | 6 | 16,770 | 4.701e-05 | 0.011343 | 8.595 |
| GO:0016020 | membrane | cellular_component | 203 | 1,626 | 1,622 | 16,770 | 5.510e-05 | 0.011633 | 1.291 |
| GO:0006099 | tricarboxylic acid cycle | biological_process | 13 | 1,626 | 41 | 16,770 | 8.362e-05 | 0.015692 | 3.270 |
| GO:0009506 | plasmodesma | cellular_component | 81 | 1,626 | 552 | 16,770 | 9.714e-05 | 0.016406 | 1.513 |
| GO:0070403 | NAD+ binding | molecular_function | 8 | 1,626 | 18 | 16,770 | 1.379e-04 | 0.02044 | 4.584 |
| GO:0004842 | ubiquitin-protein transferase activity | molecular_function | 47 | 1,626 | 281 | 16,770 | 1.452e-04 | 0.02044 | 1.725 |
| GO:0005794 | Golgi apparatus | cellular_component | 92 | 1,626 | 658 | 16,770 | 1.976e-04 | 0.025669 | 1.442 |
| GO:0003993 | acid phosphatase activity | molecular_function | 11 | 1,626 | 34 | 16,770 | 2.394e-04 | 0.026615 | 3.337 |
| GO:0015297 | antiporter activity | molecular_function | 21 | 1,626 | 95 | 16,770 | 2.481e-04 | 0.026615 | 2.280 |
| GO:0006470 | protein dephosphorylation | biological_process | 16 | 1,626 | 63 | 16,770 | 2.521e-04 | 0.026615 | 2.619 |
| GO:0006086 | acetyl-CoA biosynthetic process from pyruvate | biological_process | 6 | 1,626 | 12 | 16,770 | 4.543e-04 | 0.045139 | 5.157 |

Supplementary Table S3. Significant GO enrichment results for the antiquewhite4 (AW4) WGCNA module.

Only terms with q < 0.05 are included. Significant terms are mainly related to transmembrane transport, mitochondrial transport, organic-acid transport, ion transport and transporter activities.

| **GO ID** | **Term** | **Category** | **List hits** | **List total** | **Background hits** | **Background total** | **P value** | **q value** | **Enrichment score** |
| --- | --- | --- | --- | --- | --- | --- | --- | --- | --- |
| GO:0015117 | thiosulfate transmembrane transporter activity | molecular_function | 2 | 102 | 5 | 16,770 | 3.620e-04 | 0.027828 | 65.765 |
| GO:0015709 | thiosulfate transport | biological_process | 2 | 102 | 5 | 16,770 | 3.620e-04 | 0.027828 | 65.765 |
| GO:0071422 | succinate transmembrane transport | biological_process | 2 | 102 | 5 | 16,770 | 3.620e-04 | 0.027828 | 65.765 |
| GO:0015131 | oxaloacetate transmembrane transporter activity | molecular_function | 2 | 102 | 6 | 16,770 | 5.408e-04 | 0.027828 | 54.804 |
| GO:0015729 | oxaloacetate transport | biological_process | 2 | 102 | 6 | 16,770 | 5.408e-04 | 0.027828 | 54.804 |
| GO:0047334 | diphosphate-fructose-6-phosphate 1-phosphotransferase activity | molecular_function | 2 | 102 | 6 | 16,770 | 5.408e-04 | 0.027828 | 54.804 |
| GO:0005310 | dicarboxylic acid transmembrane transporter activity | molecular_function | 2 | 102 | 7 | 16,770 | 7.542e-04 | 0.027828 | 46.975 |
| GO:0015116 | sulfate transmembrane transporter activity | molecular_function | 2 | 102 | 7 | 16,770 | 7.542e-04 | 0.027828 | 46.975 |
| GO:0015141 | succinate transmembrane transporter activity | molecular_function | 2 | 102 | 7 | 16,770 | 7.542e-04 | 0.027828 | 46.975 |
| GO:0071423 | malate transmembrane transport | biological_process | 2 | 102 | 7 | 16,770 | 7.542e-04 | 0.027828 | 46.975 |
| GO:0015140 | malate transmembrane transporter activity | molecular_function | 2 | 102 | 8 | 16,770 | 0.001002 | 0.030798 | 41.103 |
| GO:0017077 | oxidative phosphorylation uncoupler activity | molecular_function | 2 | 102 | 8 | 16,770 | 0.001002 | 0.030798 | 41.103 |
| GO:2000038 | regulation of stomatal complex development | biological_process | 2 | 102 | 9 | 16,770 | 0.001283 | 0.036406 | 36.536 |
| GO:0000774 | adenyl-nucleotide exchange factor activity | molecular_function | 2 | 102 | 10 | 16,770 | 0.001597 | 0.039283 | 32.882 |
| GO:0008272 | sulfate transport | biological_process | 2 | 102 | 10 | 16,770 | 0.001597 | 0.039283 | 32.882 |
| GO:0005249 | voltage-gated potassium channel activity | molecular_function | 2 | 102 | 11 | 16,770 | 0.001944 | 0.042197 | 29.893 |
| GO:0006839 | mitochondrial transport | biological_process | 2 | 102 | 11 | 16,770 | 0.001944 | 0.042197 | 29.893 |
| GO:0003872 | 6-phosphofructokinase activity | molecular_function | 2 | 102 | 12 | 16,770 | 0.002324 | 0.047634 | 27.402 |

Supplementary Table S4. Significant KEGG enrichment results for the pink WGCNA module.

Only pathways with q < 0.05 are included. Arginine and proline metabolism, glutathione metabolism, glycolysis/gluconeogenesis and oxidative phosphorylation do not meet the q < 0.05 criterion and are therefore not listed as significant.

| **KEGG ID** | **Pathway** | **Level 1** | **Level 2** | **List hits** | **List total** | **Background hits** | **Background total** | **P value** | **q value** | **Enrichment score** |
| --- | --- | --- | --- | --- | --- | --- | --- | --- | --- | --- |
| dct00770 | Pantothenate and CoA biosynthesis | Metabolism | Metabolism of cofactors and vitamins | 15 | 556 | 31 | 4,666 | 5.580e-07 | 6.920e-05 | 4.061 |
| dct00020 | Citrate cycle (TCA cycle) | Metabolism | Carbohydrate metabolism | 16 | 556 | 44 | 4,666 | 2.250e-05 | 0.001396 | 3.052 |
| dct00561 | Glycerolipid metabolism | Metabolism | Lipid metabolism | 19 | 556 | 71 | 4,666 | 4.480e-04 | 0.016201 | 2.246 |
| dct04146 | Peroxisome | Cellular Processes | Transport and catabolism | 21 | 556 | 83 | 4,666 | 5.230e-04 | 0.016201 | 2.123 |
| dct00071 | Fatty acid degradation | Metabolism | Lipid metabolism | 13 | 556 | 43 | 4,666 | 0.001039 | 0.025756 | 2.537 |
| dct04136 | Autophagy - other | Cellular Processes | Transport and catabolism | 11 | 556 | 36 | 4,666 | 0.002277 | 0.045863 | 2.564 |
| dct00945 | Stilbenoid, diarylheptanoid and gingerol biosynthesis | Metabolism | Biosynthesis of other secondary metabolites | 7 | 556 | 18 | 4,666 | 0.003207 | 0.045863 | 3.264 |
| dct00620 | Pyruvate metabolism | Metabolism | Carbohydrate metabolism | 20 | 556 | 89 | 4,666 | 0.003323 | 0.045863 | 1.886 |
| dct00280 | Valine, leucine and isoleucine degradation | Metabolism | Amino acid metabolism | 11 | 556 | 38 | 4,666 | 0.003653 | 0.045863 | 2.429 |
| dct00565 | Ether lipid metabolism | Metabolism | Lipid metabolism | 8 | 556 | 23 | 4,666 | 0.003699 | 0.045863 | 2.919 |
| dct00270 | Cysteine and methionine metabolism | Metabolism | Amino acid metabolism | 20 | 556 | 91 | 4,666 | 0.004356 | 0.049108 | 1.844 |

Supplementary Table S5. KEGG pathways showing nominal enrichment in the antiquewhite4 (AW4) module.

These pathways have raw P < 0.05, but none remains significant after multiple-testing correction (all q = 0.159573). They should not be described as significantly enriched pathways. 'Steroid biosynthesis' is not equivalent to 'brassinosteroid biosynthesis'.

| **KEGG ID** | **Pathway** | **Level 1** | **Level 2** | **List hits** | **List total** | **Background hits** | **Background total** | **P value** | **q value** | **Enrichment score** |
| --- | --- | --- | --- | --- | --- | --- | --- | --- | --- | --- |
| dct00500 | Starch and sucrose metabolism | Metabolism | Carbohydrate metabolism | 4 | 35 | 114 | 4,666 | 0.00987 | 0.159573 | 4.678 |
| dct04016 | MAPK signaling pathway - plant | Environmental Information Processing | Signal transduction | 4 | 35 | 129 | 4,666 | 0.015042 | 0.159573 | 4.134 |
| dct00100 | Steroid biosynthesis | Metabolism | Lipid metabolism | 2 | 35 | 27 | 4,666 | 0.017061 | 0.159573 | 9.875 |
| dct00999 | Biosynthesis of various plant secondary metabolites | Metabolism | Biosynthesis of other secondary metabolites | 2 | 35 | 42 | 4,666 | 0.039024 | 0.159573 | 6.348 |
| dct00940 | Phenylpropanoid biosynthesis | Metabolism | Biosynthesis of other secondary metabolites | 3 | 35 | 101 | 4,666 | 0.039063 | 0.159573 | 3.960 |
| dct00860 | Porphyrin metabolism | Metabolism | Metabolism of cofactors and vitamins | 2 | 35 | 44 | 4,666 | 0.04248 | 0.159573 | 6.060 |
| dct00130 | Ubiquinone and other terpenoid-quinone biosynthesis | Metabolism | Metabolism of cofactors and vitamins | 2 | 35 | 46 | 4,666 | 0.046048 | 0.159573 | 5.796 |
| dct00010 | Glycolysis / Gluconeogenesis | Metabolism | Carbohydrate metabolism | 3 | 35 | 108 | 4,666 | 0.046192 | 0.159573 | 3.703 |
| dct00030 | Pentose phosphate pathway | Metabolism | Carbohydrate metabolism | 2 | 35 | 47 | 4,666 | 0.047872 | 0.159573 | 5.673 |

Supplementary Table S6. Functional annotation and expression concordance of the 10 genes selected for qRT-PCR validation.

Expression concordance was evaluated using log2 fold changes at 4 d relative to 0 d under EBR treatment. Pearson correlation between RNA-seq and qRT-PCR was r = 0.950 (P < 0.001; Supplementary Figure S2).

| **Gene ID** | **Functional annotation** | **RNA-seq log2FC (4 d vs 0 d)** | **qRT-PCR log2FC (4 d vs 0 d)** | **RNA-seq trend** | **qRT-PCR trend** |
| --- | --- | --- | --- | --- | --- |
| LOC110094765 | Phosphoenolpyruvate carboxylase 2 | -1.842 | -2.457 | Down | Down |
| LOC110106494 | Chlorophyll a-b binding protein 3, chloroplastic | -4.631 | -6.232 | Down | Down |
| LOC110092540 | Serine--glyoxylate aminotransferase | 1.165 | -0.112 | Up | Slightly down |
| LOC110103967 | Pyruvate, phosphate dikinase, chloroplastic | 0.009 | -0.669 | Little change | Down |
| LOC110110265 | Glycine dehydrogenase (decarboxylating), mitochondrial | -0.771 | -0.447 | Down | Down |
| LOC110114476 | Serine hydroxymethyltransferase, mitochondrial | -0.630 | -0.510 | Down | Down |
| LOC110112846 | Glycerate dehydrogenase | -0.925 | -0.774 | Down | Down |
| LOC110097304 | Probable glutathione S-transferase parA | 3.785 | 5.907 | Up | Up |
| LOC110104806 | Fructose-bisphosphate aldolase 1, chloroplastic | -1.540 | -0.181 | Down | Slightly down |
| LOC110113225 | GTP-binding protein SAR1A-like | 1.086 | 1.179 | Up | Up |

Note: Gene identities and annotations are reported for the 10 qRT-PCR-validated genes. The correlation analysis uses matched 4 d-versus-0 d expression changes from the two methods; qRT-PCR fold changes were derived from the archived mean relative-expression values represented in Supplementary Figure S1.

# Supplementary Figures

Supplementary Figure S1. qRT-PCR expression profiles of 10 representative genes used for qRT-PCR validation under the selected 2.0 mg/L EBR treatment. Relative expression was measured at 0, 2, 4, and 6 d during chilling and at 9 d, corresponding to the first day of recovery. Values are shown as mean ± SD of three biological replicates.

Supplementary Figure S2. Correlation between RNA-seq and qRT-PCR expression changes for the 10 representative genes. Pearson correlation was calculated using log2 fold changes at 4 d relative to 0 d under EBR treatment. Each point represents one gene (r = 0.950, P < 0.001).
